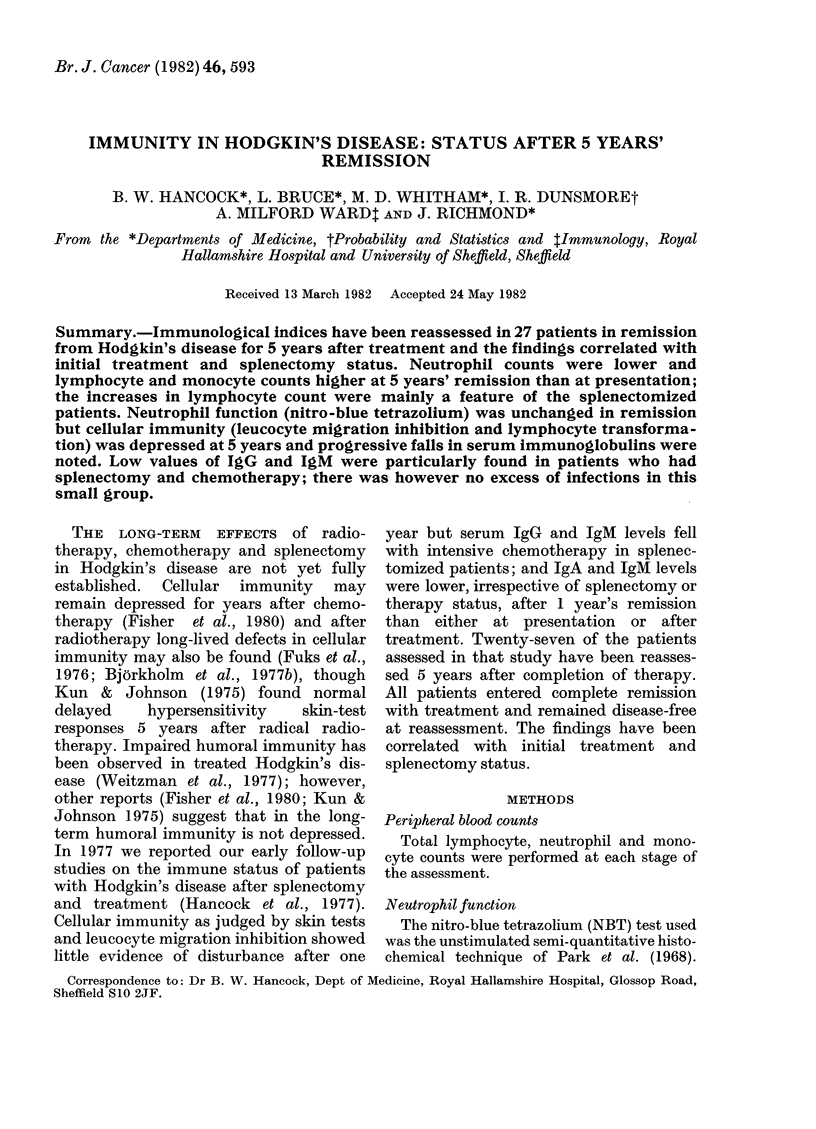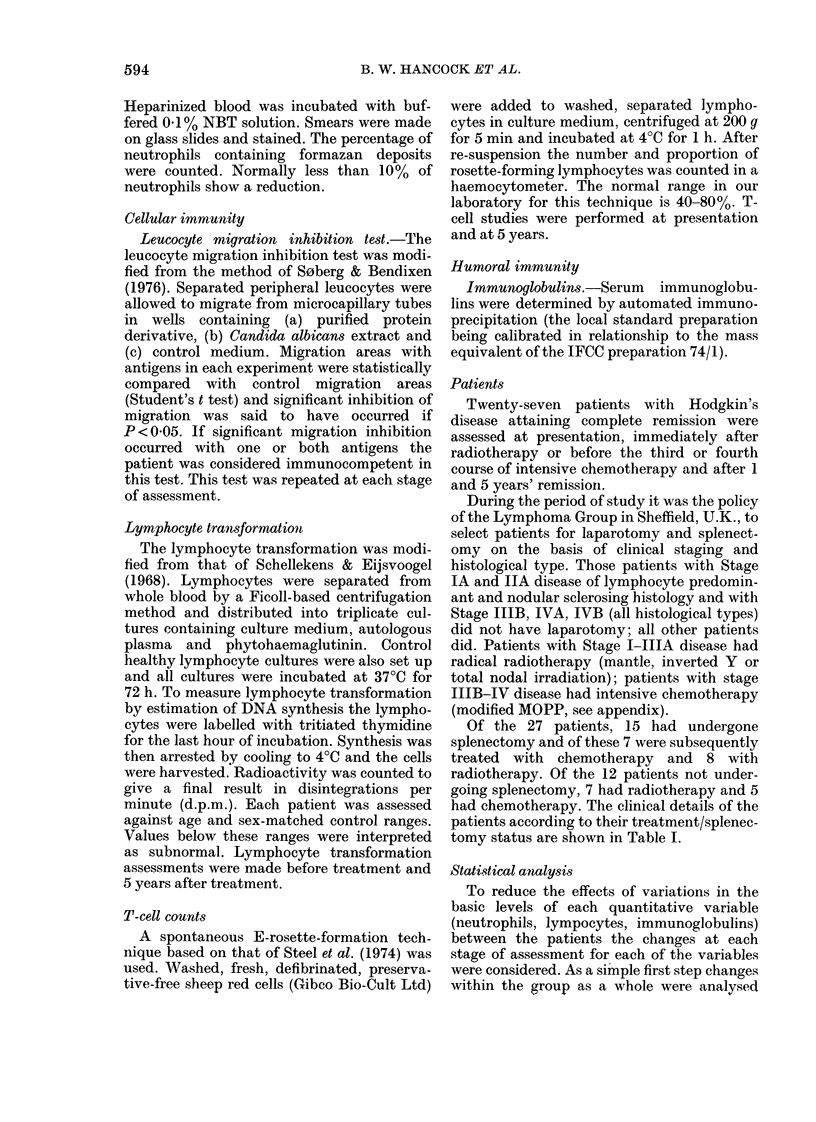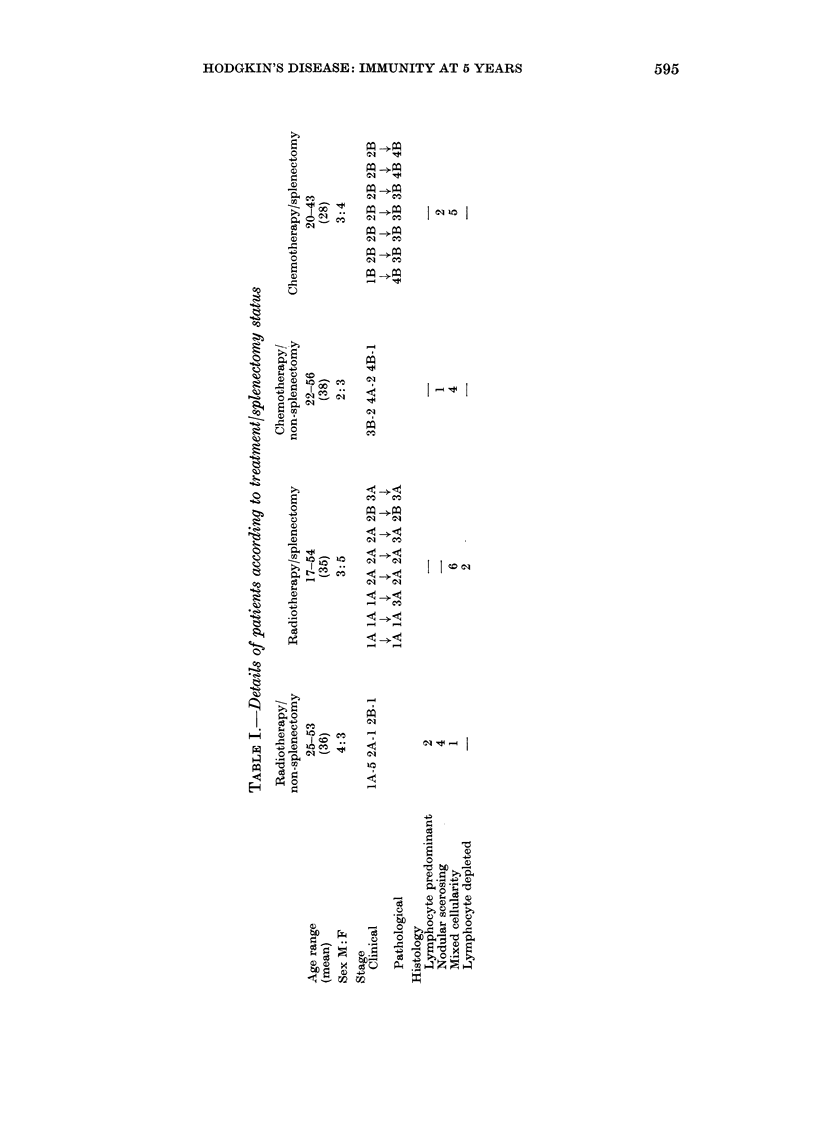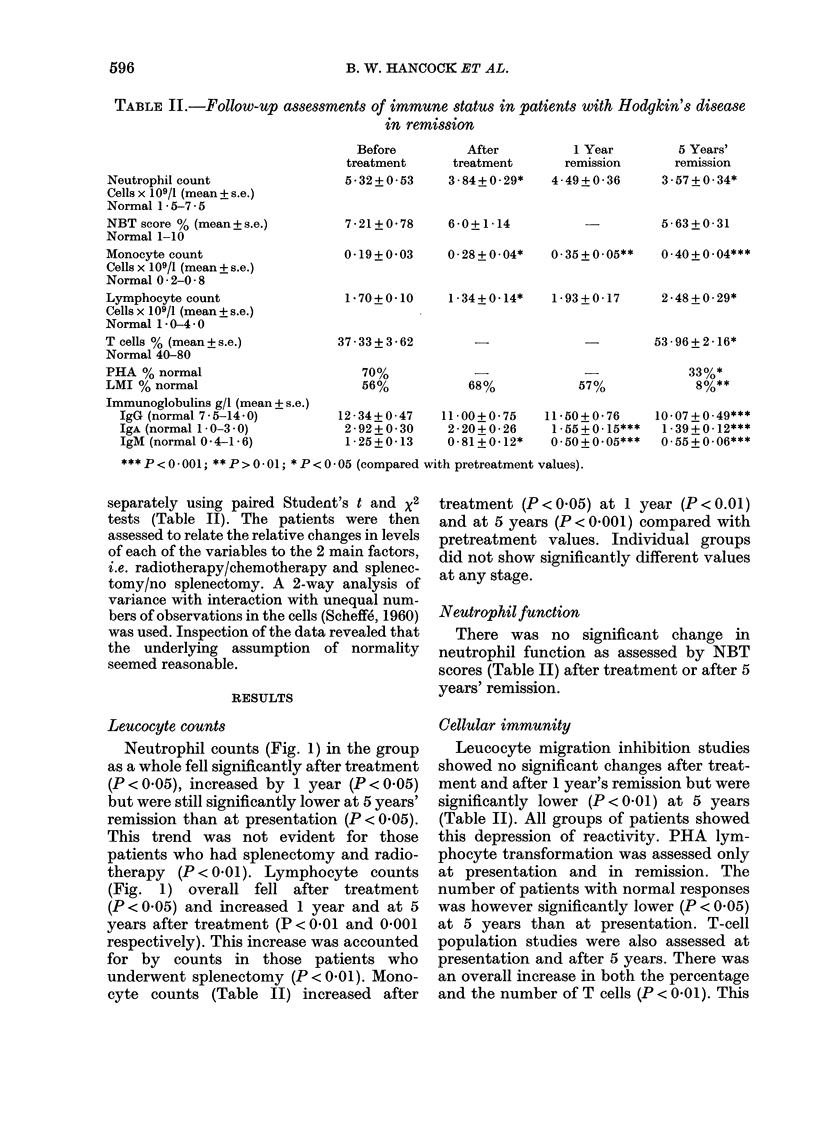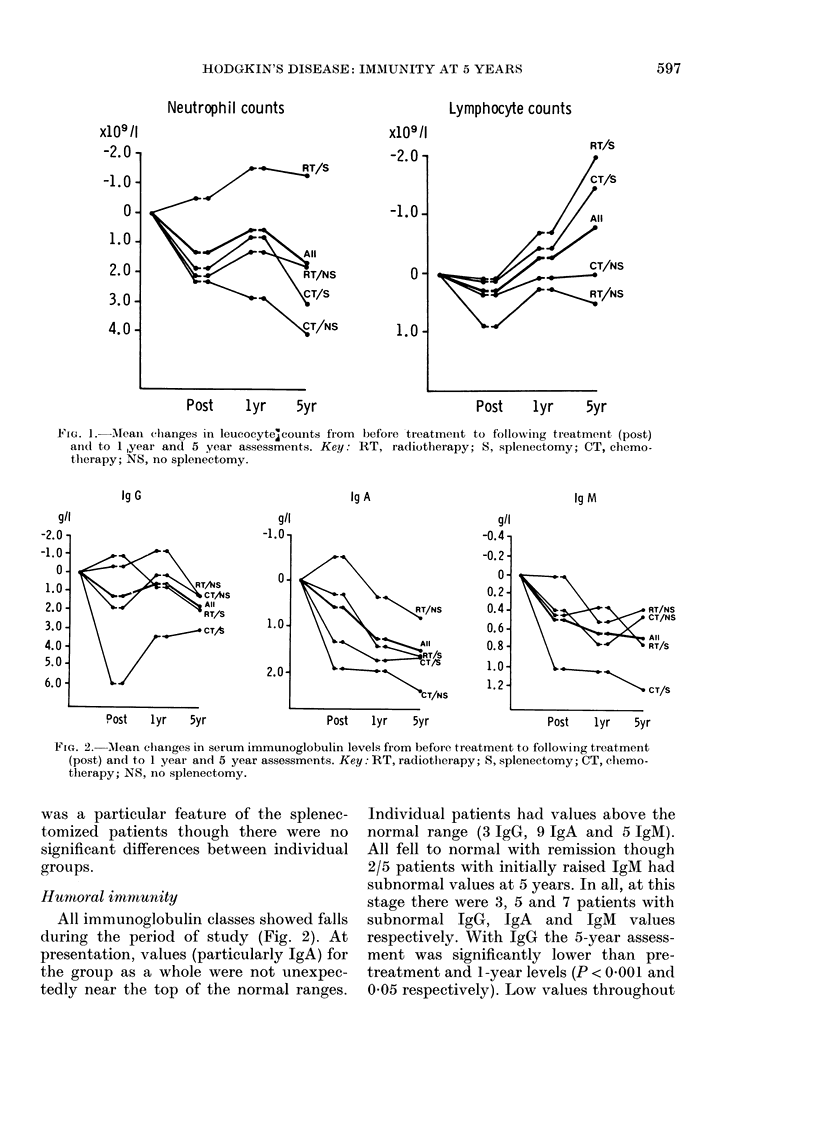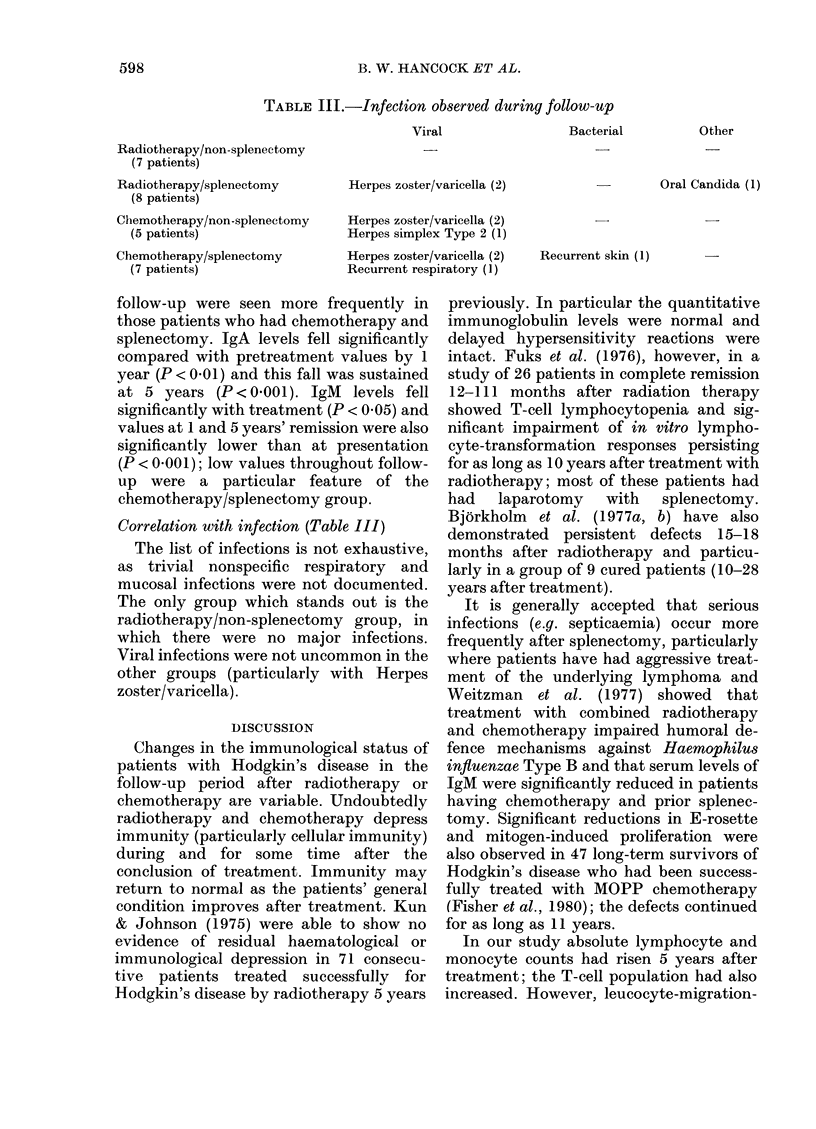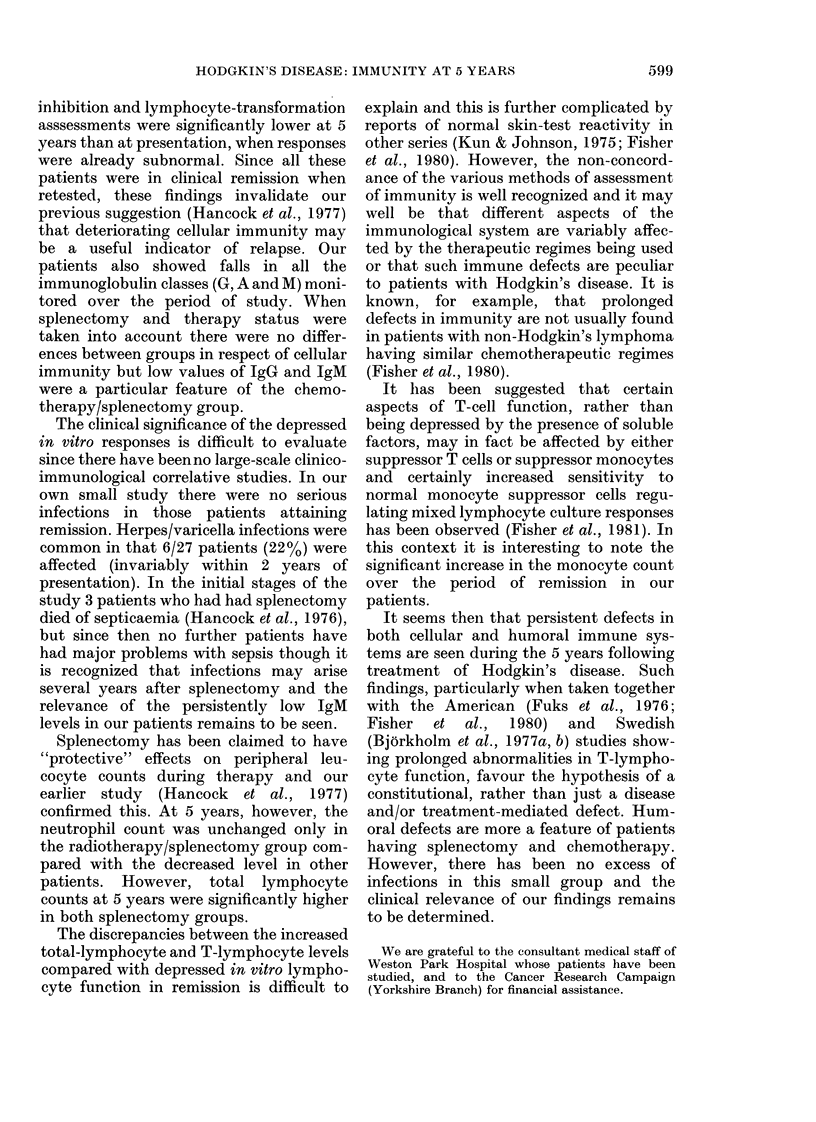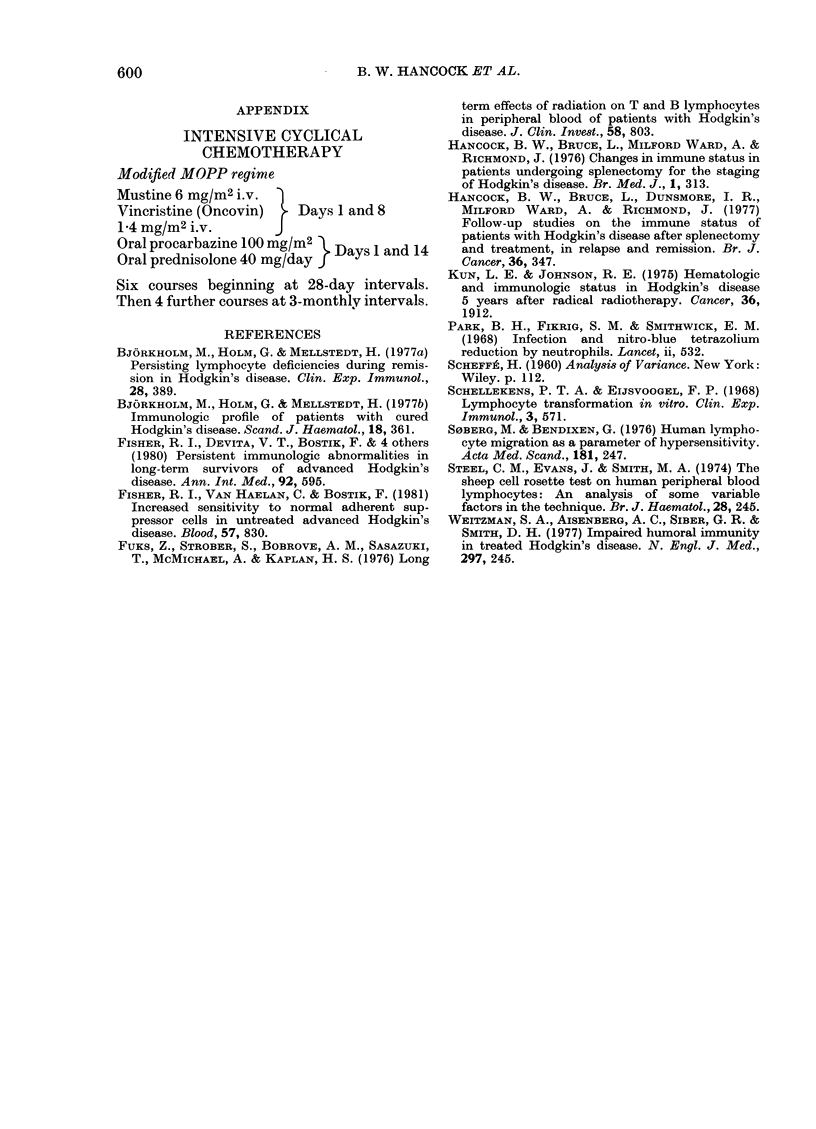# Immunity in Hodgkin's disease: status after 5 years' remission.

**DOI:** 10.1038/bjc.1982.243

**Published:** 1982-10

**Authors:** B. W. Hancock, L. Bruce, M. D. Whitham, I. R. Dunsmore, A. M. Ward, J. Richmond

## Abstract

Immunological indices have been reassessed in 27 patients in remission from Hodgkin's disease for 5 years after treatment and the findings correlated with initial treatment and splenectomy status. Neutrophil counts were lower and lymphocyte and monocyte counts higher at 5 years' remission than at presentation; the increases in lymphocyte count were mainly a feature of the splenectomized patients. Neutrophil function (nitro-blue tetrazolium) was unchanged in remission but cellular immunity (leucocyte migration inhibition and lymphocyte transformation) was depressed at 5 years and progressive falls in serum immunoglobulins were noted. Low values of IgG and IgM were particularly found in patients who had splenectomy and chemotherapy; there was however no excess of infections in this small group.


*Br. J. Cancer* (1982) **46**, 593

### IMMUNITY IN HODGKIN'S DISEASE: STATUS AFTER 5 YEARS' REMISSION

B. W. HANCOCK*, L. BRUCE*, M. D. WHITHAM*, I. R. DUNSMORE† A. MILFORD WARD‡ AND J. RICHMOND*

*From the *Departments of Medicine, †Probability and Statistics and ‡Immunology, Royal Hallamshire Hospital and University of Sheffield, Sheffield*

Received 13 March 1982 Accepted 24 May 1982

**Summary.—Immunological indices have been reassessed in 27 patients in remission from Hodgkin's disease for 5 years after treatment and the findings correlated with initial treatment and splenectomy status. Neutrophil counts were lower and lymphocyte and monocyte counts higher at 5 years' remission than at presentation; the increases in lymphocyte count were mainly a feature of the splenectomized patients. Neutrophil function (nitro-blue tetrazolium) was unchanged in remission but cellular immunity (leucocyte migration inhibition and lymphocyte transformation) was depressed at 5 years and progressive falls in serum immunoglobulins were noted. Low values of IgG and IgM were particularly found in patients who had splenectomy and chemotherapy; there was however no excess of infections in this small group.**

THE LONG-TERM EFFECTS of radiotherapy, chemotherapy and splenectomy in Hodgkin's disease are not yet fully established. Cellular immunity may remain depressed for years after chemotherapy (Fisher *et al.*, 1980) and after radiotherapy long-lived defects in cellular immunity may also be found (Fuks *et al.*, 1976; Björkholm *et al.*, 1977*b*), though Kun & Johnson (1975) found normal delayed hypersensitivity skin-test responses 5 years after radical radiotherapy. Impaired humoral immunity has been observed in treated Hodgkin's disease (Weitzman *et al.*, 1977); however, other reports (Fisher *et al.*, 1980; Kun & Johnson 1975) suggest that in the long-term humoral immunity is not depressed. In 1977 we reported our early follow-up studies on the immune status of patients with Hodgkin's disease after splenectomy and treatment (Hancock *et al.*, 1977). Cellular immunity as judged by skin tests and leucocyte migration inhibition showed little evidence of disturbance after one year but serum IgG and IgM levels fell with intensive chemotherapy in splenectomized patients; and IgA and IgM levels were lower, irrespective of splenectomy or therapy status, after 1 year's remission than either at presentation or after treatment. Twenty-seven of the patients assessed in that study have been reassessed 5 years after completion of therapy. All patients entered complete remission with treatment and remained disease-free at reassessment. The findings have been correlated with initial treatment and splenectomy status.

### METHODS

### *Peripheral blood counts*

Total lymphocyte, neutrophil and monocyte counts were performed at each stage of the assessment.

### *Neutrophil function*

The nitro-blue tetrazolium (NBT) test used was the unstimulated semi-quantitative histochemical technique of Park *et al.* (1968).

Correspondence to: Dr B. W. Hancock, Dept of Medicine, Royal Hallamshire Hospital, Glossop Road, Sheffield S10 2JF.

Heparinized blood was incubated with buffered 0·1% NBT solution. Smears were made on glass slides and stained. The percentage of neutrophils containing formazan deposits were counted. Normally less than 10% of neutrophils show a reduction.

*Cellular immunity*

*Leucocyte migration inhibition test.*—The leucocyte migration inhibition test was modified from the method of Søberg & Bendixen (1976). Separated peripheral leucocytes were allowed to migrate from microcapillary tubes in wells containing (a) purified protein derivative, (b) *Candida albicans* extract and (c) control medium. Migration areas with antigens in each experiment were statistically compared with control migration areas (Student's *t* test) and significant inhibition of migration was said to have occurred if $P < 0{\cdot}05$. If significant migration inhibition occurred with one or both antigens the patient was considered immunocompetent in this test. This test was repeated at each stage of assessment.

*Lymphocyte transformation*

The lymphocyte transformation was modified from that of Schellekens & Eijsvoogel (1968). Lymphocytes were separated from whole blood by a Ficoll-based centrifugation method and distributed into triplicate cultures containing culture medium, autologous plasma and phytohaemaglutinin. Control healthy lymphocyte cultures were also set up and all cultures were incubated at 37°C for 72 h. To measure lymphocyte transformation by estimation of DNA synthesis the lymphocytes were labelled with tritiated thymidine for the last hour of incubation. Synthesis was then arrested by cooling to 4°C and the cells were harvested. Radioactivity was counted to give a final result in disintegrations per minute (d.p.m.). Each patient was assessed against age and sex-matched control ranges. Values below these ranges were interpreted as subnormal. Lymphocyte transformation assessments were made before treatment and 5 years after treatment.

*T-cell counts*

A spontaneous E-rosette-formation technique based on that of Steel *et al.* (1974) was used. Washed, fresh, defibrinated, preservative-free sheep red cells (Gibco Bio-Cult Ltd) were added to washed, separated lymphocytes in culture medium, centrifuged at 200 *g* for 5 min and incubated at 4°C for 1 h. After re-suspension the number and proportion of rosette-forming lymphocytes was counted in a haemocytometer. The normal range in our laboratory for this technique is 40–80%. T-cell studies were performed at presentation and at 5 years.

*Humoral immunity*

*Immunoglobulins.*—Serum immunoglobulins were determined by automated immunoprecipitation (the local standard preparation being calibrated in relationship to the mass equivalent of the IFCC preparation 74/1).

*Patients*

Twenty-seven patients with Hodgkin's disease attaining complete remission were assessed at presentation, immediately after radiotherapy or before the third or fourth course of intensive chemotherapy and after 1 and 5 years' remission.

During the period of study it was the policy of the Lymphoma Group in Sheffield, U.K., to select patients for laparotomy and splenectomy on the basis of clinical staging and histological type. Those patients with Stage IA and IIA disease of lymphocyte predominant and nodular sclerosing histology and with Stage IIIB, IVA, IVB (all histological types) did not have laparotomy; all other patients did. Patients with Stage I–IIIA disease had radical radiotherapy (mantle, inverted Y or total nodal irradiation); patients with stage IIIB–IV disease had intensive chemotherapy (modified MOPP, see appendix).

Of the 27 patients, 15 had undergone splenectomy and of these 7 were subsequently treated with chemotherapy and 8 with radiotherapy. Of the 12 patients not undergoing splenectomy, 7 had radiotherapy and 5 had chemotherapy. The clinical details of the patients according to their treatment/splenectomy status are shown in Table I.

*Statistical analysis*

To reduce the effects of variations in the basic levels of each quantitative variable (neutrophils, lympocytes, immunoglobulins) between the patients the changes at each stage of assessment for each of the variables were considered. As a simple first step changes within the group as a whole were analysed

TABLE I.—*Details of patients according to treatment/splenectomy status*

| | Radiotherapy/ non-splenectomy | Radiotherapy/splenectomy | Chemotherapy/ non-splenectomy | Chemotherapy/splenectomy |
|---|---|---|---|---|
| Age range (mean) | 25–53 (36) | 17–54 (35) | 22–56 (38) | 20–43 (28) |
| Sex M:F | 4:3 | 3:5 | 2:3 | 3:4 |
| Stage | | | | |
| Clinical | 1A-5 2A-1 2B-1 | 1A 1A 1A 2A 2A 2A 2B 3A | 3B-2 4A-2 4B-1 | 1B 2B 2B 2B 2B 2B 2B |
| | | ↓ ↓ ↓ ↓ ↓ ↓ ↓ ↓ | | ↓ ↓ ↓ ↓ ↓ ↓ ↓ |
| Pathological | | 1A 1A 3A 2A 2A 3A 2B 3A | | 4B 3B 3B 3B 3B 4B 4B |
| Histology | | | | |
| Lymphocyte predominant | 2 | — | — | — |
| Nodular scerosing | 4 | — | 1 | 2 |
| Mixed cellularity | 1 | 6 | 4 | 5 |
| Lymphocyte depleted | — | 2 | — | — |

TABLE II.—*Follow-up assessments of immune status in patients with Hodgkin's disease in remission*

| | Before treatment | After treatment | 1 Year remission | 5 Years' remission |
|---|---|---|---|---|
| Neutrophil count<br>Cells × $10^9$/l (mean ± s.e.)<br>Normal 1·5–7·5 | 5·32 ± 0·53 | 3·84 ± 0·29* | 4·49 ± 0·36 | 3·57 ± 0·34* |
| NBT score % (mean ± s.e.)<br>Normal 1–10 | 7·21 ± 0·78 | 6·0 ± 1·14 | — | 5·63 ± 0·31 |
| Monocyte count<br>Cells × $10^9$/l (mean ± s.e.)<br>Normal 0·2–0·8 | 0·19 ± 0·03 | 0·28 ± 0·04* | 0·35 ± 0·05** | 0·40 ± 0·04*** |
| Lymphocyte count<br>Cells × $10^9$/l (mean ± s.e.)<br>Normal 1·0–4·0 | 1·70 ± 0·10 | 1·34 ± 0·14* | 1·93 ± 0·17 | 2·48 ± 0·29* |
| T cells % (mean ± s.e.)<br>Normal 40–80 | 37·33 ± 3·62 | — | — | 53·96 ± 2·16* |
| PHA % normal | 70% | — | — | 33%* |
| LMI % normal | 56% | 68% | 57% | 8%** |
| Immunoglobulins g/l (mean ± s.e.) | | | | |
| IgG (normal 7·5–14·0) | 12·34 ± 0·47 | 11·00 ± 0·75 | 11·50 ± 0·76 | 10·07 ± 0·49*** |
| IgA (normal 1·0–3·0) | 2·92 ± 0·30 | 2·20 ± 0·26 | 1·55 ± 0·15*** | 1·39 ± 0·12*** |
| IgM (normal 0·4–1·6) | 1·25 ± 0·13 | 0·81 ± 0·12* | 0·50 ± 0·05*** | 0·55 ± 0·06*** |

*** $P < 0·001$; ** $P > 0·01$; * $P < 0·05$ (compared with pretreatment values).

separately using paired Student's $t$ and $\chi^2$ tests (Table II). The patients were then assessed to relate the relative changes in levels of each of the variables to the 2 main factors, *i.e.* radiotherapy/chemotherapy and splenectomy/no splenectomy. A 2-way analysis of variance with interaction with unequal numbers of observations in the cells (Scheffé, 1960) was used. Inspection of the data revealed that the underlying assumption of normality seemed reasonable.

### RESULTS

### *Leucocyte counts*

Neutrophil counts (Fig. 1) in the group as a whole fell significantly after treatment ($P < 0·05$), increased by 1 year ($P < 0·05$) but were still significantly lower at 5 years' remission than at presentation ($P < 0·05$). This trend was not evident for those patients who had splenectomy and radiotherapy ($P < 0·01$). Lymphocyte counts (Fig. 1) overall fell after treatment ($P < 0·05$) and increased 1 year and at 5 years after treatment ($P < 0·01$ and 0·001 respectively). This increase was accounted for by counts in those patients who underwent splenectomy ($P < 0·01$). Monocyte counts (Table II) increased after treatment ($P < 0·05$) at 1 year ($P < 0.01$) and at 5 years ($P < 0·001$) compared with pretreatment values. Individual groups did not show significantly different values at any stage.

### *Neutrophil function*

There was no significant change in neutrophil function as assessed by NBT scores (Table II) after treatment or after 5 years' remission.

### *Cellular immunity*

Leucocyte migration inhibition studies showed no significant changes after treatment and after 1 year's remission but were significantly lower ($P < 0·01$) at 5 years (Table II). All groups of patients showed this depression of reactivity. PHA lymphocyte transformation was assessed only at presentation and in remission. The number of patients with normal responses was however significantly lower ($P < 0·05$) at 5 years than at presentation. T-cell population studies were also assessed at presentation and after 5 years. There was an overall increase in both the percentage and the number of T cells ($P < 0·01$). This

FIG. 1.—Mean changes in leucocyte counts from before treatment to following treatment (post) and to 1 year and 5 year assessments. *Key:* RT, radiotherapy; S, splenectomy; CT, chemotherapy; NS, no splenectomy.

FIG. 2.—Mean changes in serum immunoglobulin levels from before treatment to following treatment (post) and to 1 year and 5 year assessments. *Key:* RT, radiotherapy; S, splenectomy; CT, chemotherapy; NS, no splenectomy.

was a particular feature of the splenectomized patients though there were no significant differences between individual groups.

*Humoral immunity*

All immunoglobulin classes showed falls during the period of study (Fig. 2). At presentation, values (particularly IgA) for the group as a whole were not unexpectedly near the top of the normal ranges. Individual patients had values above the normal range (3 IgG, 9 IgA and 5 IgM). All fell to normal with remission though 2/5 patients with initially raised IgM had subnormal values at 5 years. In all, at this stage there were 3, 5 and 7 patients with subnormal IgG, IgA and IgM values respectively. With IgG the 5-year assessment was significantly lower than pretreatment and 1-year levels ($P < 0{\cdot}001$ and $0{\cdot}05$ respectively). Low values throughout

TABLE III.—*Infection observed during follow-up*

| | Viral | Bacterial | Other |
|---|---|---|---|
| Radiotherapy/non-splenectomy (7 patients) | — | — | — |
| Radiotherapy/splenectomy (8 patients) | Herpes zoster/varicella (2) | — | Oral Candida (1) |
| Chemotherapy/non-splenectomy (5 patients) | Herpes zoster/varicella (2)<br>Herpes simplex Type 2 (1) | — | — |
| Chemotherapy/splenectomy (7 patients) | Herpes zoster/varicella (2)<br>Recurrent respiratory (1) | Recurrent skin (1) | — |

follow-up were seen more frequently in those patients who had chemotherapy and splenectomy. IgA levels fell significantly compared with pretreatment values by 1 year ($P < 0{\cdot}01$) and this fall was sustained at 5 years ($P < 0{\cdot}001$). IgM levels fell significantly with treatment ($P < 0{\cdot}05$) and values at 1 and 5 years' remission were also significantly lower than at presentation ($P < 0{\cdot}001$); low values throughout follow-up were a particular feature of the chemotherapy/splenectomy group.

*Correlation with infection (Table III)*

The list of infections is not exhaustive, as trivial nonspecific respiratory and mucosal infections were not documented. The only group which stands out is the radiotherapy/non-splenectomy group, in which there were no major infections. Viral infections were not uncommon in the other groups (particularly with Herpes zoster/varicella).

### DISCUSSION

Changes in the immunological status of patients with Hodgkin's disease in the follow-up period after radiotherapy or chemotherapy are variable. Undoubtedly radiotherapy and chemotherapy depress immunity (particularly cellular immunity) during and for some time after the conclusion of treatment. Immunity may return to normal as the patients' general condition improves after treatment. Kun & Johnson (1975) were able to show no evidence of residual haematological or immunological depression in 71 consecutive patients treated successfully for Hodgkin's disease by radiotherapy 5 years previously. In particular the quantitative immunoglobulin levels were normal and delayed hypersensitivity reactions were intact. Fuks *et al.* (1976), however, in a study of 26 patients in complete remission 12–111 months after radiation therapy showed T-cell lymphocytopenia and significant impairment of *in vitro* lymphocyte-transformation responses persisting for as long as 10 years after treatment with radiotherapy; most of these patients had had laparotomy with splenectomy. Björkholm *et al.* (1977*a*, *b*) have also demonstrated persistent defects 15–18 months after radiotherapy and particularly in a group of 9 cured patients (10–28 years after treatment).

It is generally accepted that serious infections (*e.g.* septicaemia) occur more frequently after splenectomy, particularly where patients have had aggressive treatment of the underlying lymphoma and Weitzman *et al.* (1977) showed that treatment with combined radiotherapy and chemotherapy impaired humoral defence mechanisms against *Haemophilus influenzae* Type B and that serum levels of IgM were significantly reduced in patients having chemotherapy and prior splenectomy. Significant reductions in E-rosette and mitogen-induced proliferation were also observed in 47 long-term survivors of Hodgkin's disease who had been successfully treated with MOPP chemotherapy (Fisher *et al.*, 1980); the defects continued for as long as 11 years.

In our study absolute lymphocyte and monocyte counts had risen 5 years after treatment; the T-cell population had also increased. However, leucocyte-migrationinhibition and lymphocyte-transformation asssessments were significantly lower at 5 years than at presentation, when responses were already subnormal. Since all these patients were in clinical remission when retested, these findings invalidate our previous suggestion (Hancock *et al.*, 1977) that deteriorating cellular immunity may be a useful indicator of relapse. Our patients also showed falls in all the immunoglobulin classes (G, A and M) monitored over the period of study. When splenectomy and therapy status were taken into account there were no differences between groups in respect of cellular immunity but low values of IgG and IgM were a particular feature of the chemotherapy/splenectomy group.

The clinical significance of the depressed *in vitro* responses is difficult to evaluate since there have been no large-scale clinico-immunological correlative studies. In our own small study there were no serious infections in those patients attaining remission. Herpes/varicella infections were common in that 6/27 patients (22%) were affected (invariably within 2 years of presentation). In the initial stages of the study 3 patients who had had splenectomy died of septicaemia (Hancock *et al.*, 1976), but since then no further patients have had major problems with sepsis though it is recognized that infections may arise several years after splenectomy and the relevance of the persistently low IgM levels in our patients remains to be seen.

Splenectomy has been claimed to have "protective" effects on peripheral leucocyte counts during therapy and our earlier study (Hancock *et al.*, 1977) confirmed this. At 5 years, however, the neutrophil count was unchanged only in the radiotherapy/splenectomy group compared with the decreased level in other patients. However, total lymphocyte counts at 5 years were significantly higher in both splenectomy groups.

The discrepancies between the increased total-lymphocyte and T-lymphocyte levels compared with depressed *in vitro* lymphocyte function in remission is difficult to explain and this is further complicated by reports of normal skin-test reactivity in other series (Kun & Johnson, 1975; Fisher *et al.*, 1980). However, the non-concordance of the various methods of assessment of immunity is well recognized and it may well be that different aspects of the immunological system are variably affected by the therapeutic regimes being used or that such immune defects are peculiar to patients with Hodgkin's disease. It is known, for example, that prolonged defects in immunity are not usually found in patients with non-Hodgkin's lymphoma having similar chemotherapeutic regimes (Fisher *et al.*, 1980).

It has been suggested that certain aspects of T-cell function, rather than being depressed by the presence of soluble factors, may in fact be affected by either suppressor T cells or suppressor monocytes and certainly increased sensitivity to normal monocyte suppressor cells regulating mixed lymphocyte culture responses has been observed (Fisher *et al.*, 1981). In this context it is interesting to note the significant increase in the monocyte count over the period of remission in our patients.

It seems then that persistent defects in both cellular and humoral immune systems are seen during the 5 years following treatment of Hodgkin's disease. Such findings, particularly when taken together with the American (Fuks *et al.*, 1976; Fisher *et al.*, 1980) and Swedish (Björkholm *et al.*, 1977*a*, *b*) studies showing prolonged abnormalities in T-lymphocyte function, favour the hypothesis of a constitutional, rather than just a disease and/or treatment-mediated defect. Humoral defects are more a feature of patients having splenectomy and chemotherapy. However, there has been no excess of infections in this small group and the clinical relevance of our findings remains to be determined.

We are grateful to the consultant medical staff of Weston Park Hospital whose patients have been studied, and to the Cancer Research Campaign (Yorkshire Branch) for financial assistance.

### APPENDIX

### INTENSIVE CYCLICAL CHEMOTHERAPY

*Modified MOPP regime*

| | |
|---|---|
| Mustine 6 mg/m$^2$ i.v.<br>Vincristine (Oncovin)<br>1·4 mg/m$^2$ i.v. | Days 1 and 8 |
| Oral procarbazine 100 mg/m$^2$<br>Oral prednisolone 40 mg/day | Days 1 and 14 |

Six courses beginning at 28-day intervals. Then 4 further courses at 3-monthly intervals.

### REFERENCES

BJÖRKHOLM, M., HOLM, G. & MELLSTEDT, H. (1977*a*) Persisting lymphocyte deficiencies during remission in Hodgkin's disease. *Clin. Exp. Immunol.*, **28**, 389.

BJÖRKHOLM, M., HOLM, G. & MELLSTEDT, H. (1977*b*) Immunologic profile of patients with cured Hodgkin's disease. *Scand. J. Haematol.*, **18**, 361.

FISHER, R. I., DEVITA, V. T., BOSTIK, F. & 4 others (1980) Persistent immunologic abnormalities in long-term survivors of advanced Hodgkin's disease. *Ann. Int. Med.*, **92**, 595.

FISHER, R. I., VAN HAELAN, C. & BOSTIK, F. (1981) Increased sensitivity to normal adherent suppressor cells in untreated advanced Hodgkin's disease. *Blood*, **57**, 830.

FUKS, Z., STROBER, S., BOBROVE, A. M., SASAZUKI, T., MCMICHAEL, A. & KAPLAN, H. S. (1976) Long term effects of radiation on T and B lymphocytes in peripheral blood of patients with Hodgkin's disease. *J. Clin. Invest.*, **58**, 803.

HANCOCK, B. W., BRUCE, L., MILFORD WARD, A. & RICHMOND, J. (1976) Changes in immune status in patients undergoing splenectomy for the staging of Hodgkin's disease. *Br. Med. J.*, **1**, 313.

HANCOCK, B. W., BRUCE, L., DUNSMORE, I. R., MILFORD WARD, A. & RICHMOND, J. (1977) Follow-up studies on the immune status of patients with Hodgkin's disease after splenectomy and treatment, in relapse and remission. *Br. J. Cancer*, **36**, 347.

KUN, L. E. & JOHNSON, R. E. (1975) Hematologic and immunologic status in Hodgkin's disease 5 years after radical radiotherapy. *Cancer*, **36**, 1912.

PARK, B. H., FIKRIG, S. M. & SMITHWICK, E. M. (1968) Infection and nitro-blue tetrazolium reduction by neutrophils. *Lancet*, ii, 532.

SCHEFFÉ, H. (1960) *Analysis of Variance*. New York: Wiley. p. 112.

SCHELLEKENS, P. T. A. & EIJSVOOGEL, F. P. (1968) Lymphocyte transformation *in vitro*. *Clin. Exp. Immunol.*, **3**, 571.

SØBERG, M. & BENDIXEN, G. (1976) Human lymphocyte migration as a parameter of hypersensitivity. *Acta Med. Scand.*, **181**, 247.

STEEL, C. M., EVANS, J. & SMITH, M. A. (1974) The sheep cell rosette test on human peripheral blood lymphocytes: An analysis of some variable factors in the technique. *Br. J. Haematol.*, **28**, 245.

WEITZMAN, S. A., AISENBERG, A. C., SIBER, G. R. & SMITH, D. H. (1977) Impaired humoral immunity in treated Hodgkin's disease. *N. Engl. J. Med.*, **297**, 245.